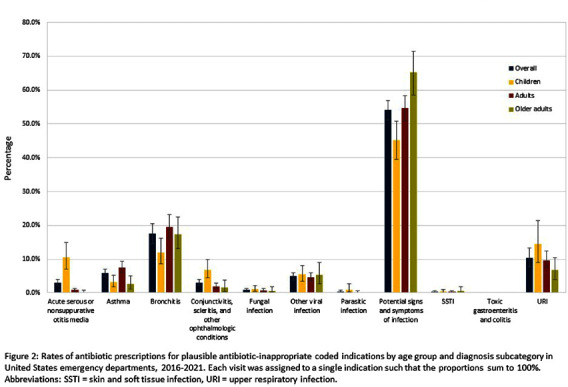# Appropriateness of Antibiotic Prescriptions in Emergency Departments in the United States, 2016-2021

**DOI:** 10.1017/ash.2024.121

**Published:** 2024-09-16

**Authors:** Joseph Ladines-Lim, Kao-Ping Chua

**Affiliations:** Michigan Medicine; University of Michigan

## Abstract

**Background:** Inappropriate antibiotic prescribing contributes to antimicrobial resistance, a global health threat. Prior studies have used ICD-9-CM codes to estimate inappropriate prescribing rates in ambulatory settings, including emergency departments (EDs), though the last national estimates date back to 2010-2015 (Hersh et al, CID 2021). Using the most recent publicly available data, we estimated inappropriate antibiotic prescribing rates in EDs across all conditions. For further characterization, we estimated rates of inappropriate antibiotic prescribing with and without codes that could be plausible indications for which antibiotics are prescribed. **Methods:** We analyzed 2016-2021 data from the National Hospital Ambulatory Medical Care Survey, a nationally representative survey of EDs, subsetting to visits with ≥1 oral antibiotic prescription. Using ICD-10-CM codes (Chua et al, BMJ 2019), we calculated proportions of visits with inappropriate antibiotic prescribing; inappropriate antibiotic prescribing and ≥1 plausible antibiotic-inappropriate indication (e.g., viral infection); and inappropriate prescribing without plausible antibiotic-inappropriate indications. Among visits with plausible antibiotic-inappropriate indications, we subcategorized these further (e.g. viral infection, ophthalmologic conditions). Among visits without plausible antibiotic-inappropriate indications, we determined the most common diagnosis codes. We conducted analyses overall and separately among children (0-17 years), adults (18-64 years), and older adults (≥65 years). **Results:** Demographic characteristics by age group are shown in Table 1. Antibiotic prescription rates overall and for children, adults, and older adults were 18.6%, 17.8%, 19.1%, and 18.0%, respectively. Inappropriate prescription rates were 27.6%, 23.7%, 29.8%, and 24.6%, respectively. Inappropriate antibiotic prescription rates with plausible indications were 14.9%, 16.7%, 15.0%, and 12.6%, while inappropriate antibiotic prescription rates without plausible indications were 12.7%, 7.0%, 14.9%, and 12.0%, respectively (Figure 1). Rates of subcategories of inappropriate prescribing with plausible antibiotic indications overall and by age group are shown in Figure 2, with the most common diagnoses for potential signs and symptoms of infection in Table 2. The most common diagnoses for inappropriate prescribing without plausible indications are in Table 3. **Conclusions:** Inappropriate antibiotic prescriptions in EDs are common with a substantial proportion without plausible conditions. Most inappropriate prescriptions with plausible antibiotic-inappropriate indications are associated with potential signs and symptoms of infection without a more definitive diagnosis code, suggesting either diagnostic uncertainty or poor coding quality. Future work should distinguish between these two possibilities to determine whether stewardship efforts should focus on educational strategies to avoid unnecessary empiric antibiotic prescribing in the setting of diagnostic uncertainty, improving coding quality, or both.

**Figure:**
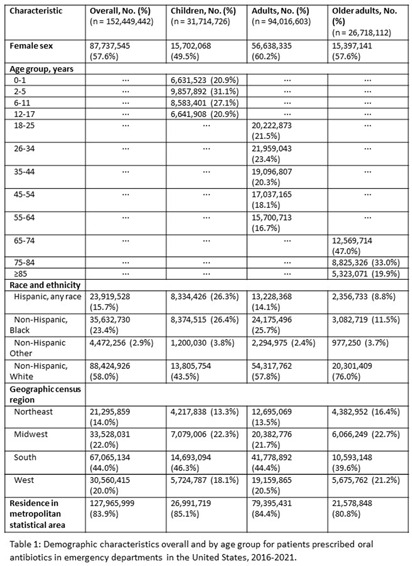

**Figure:**
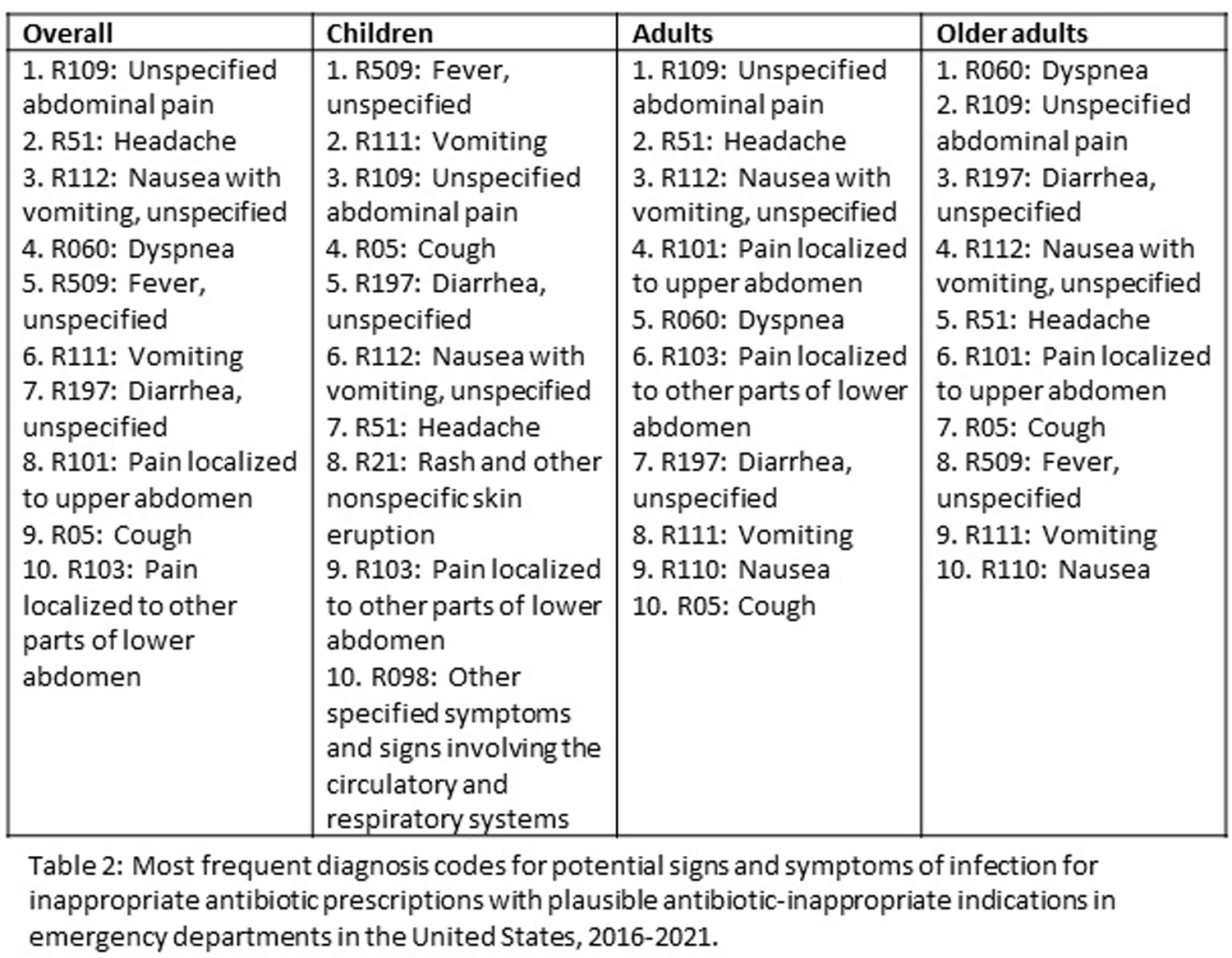

**Figure:**
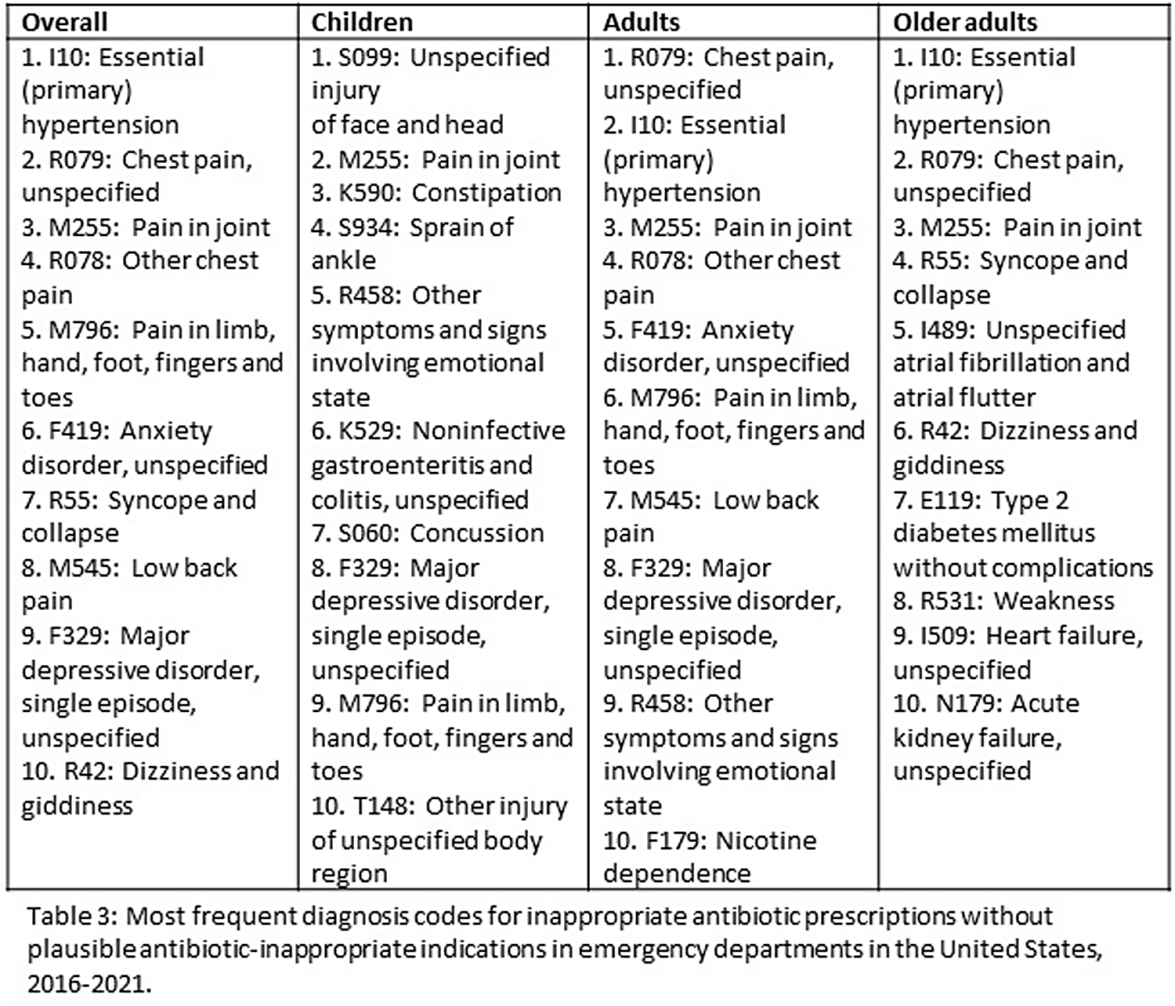

**Figure:**
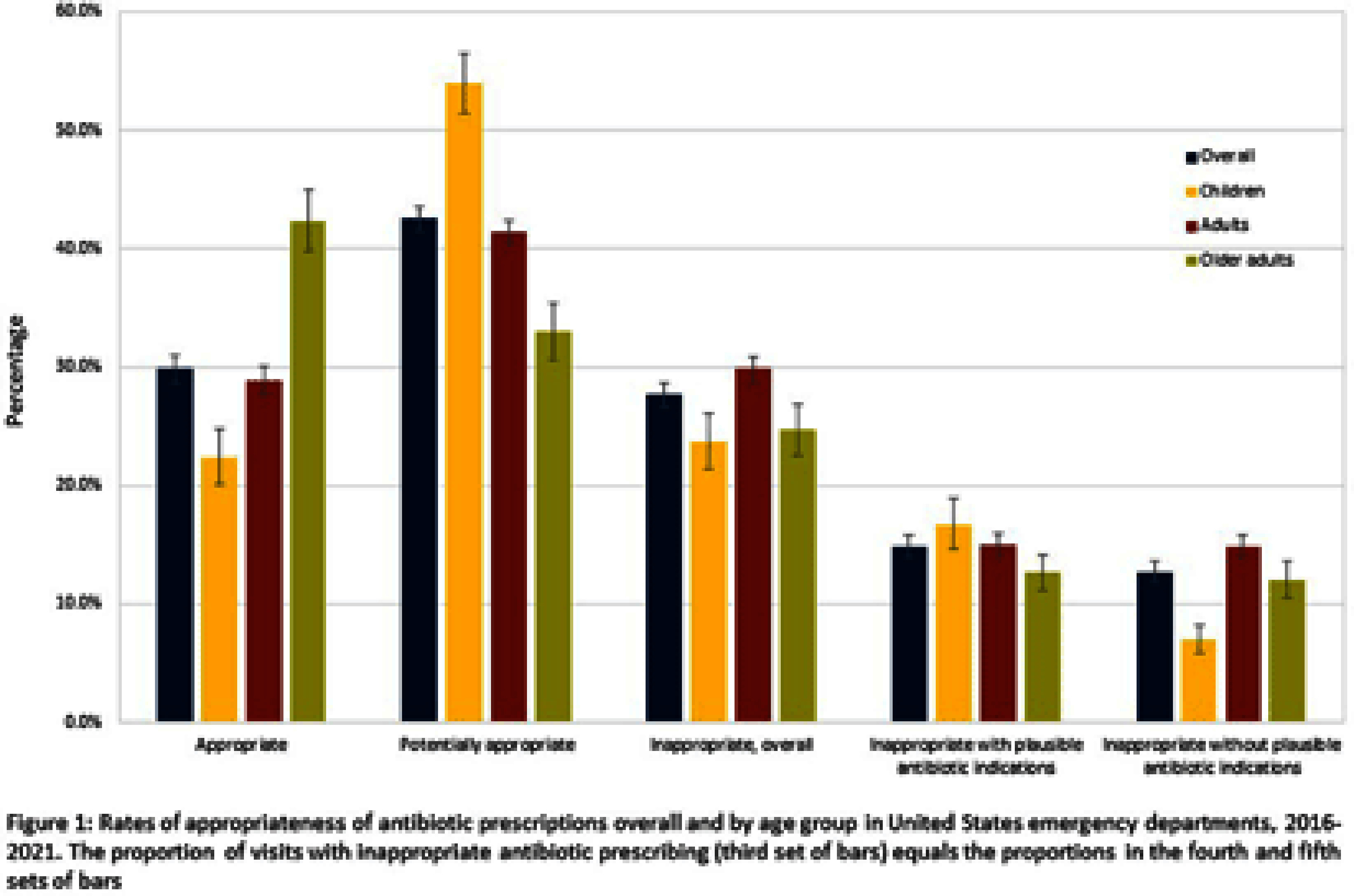

**Figure:**